# Point-of-care versus central testing of hemoglobin during large volume blood transfusion

**DOI:** 10.1186/s12871-019-0916-2

**Published:** 2019-12-27

**Authors:** Justin Herman, Brian Park, Bharat Awsare, Frances West, Denine Crittendon, Lilah Evans, Mary Harach, Julie Karp, Alexis Peedin, Marianna LaNoue, Barbara Goldsmith, Eugene Warnick, Michael Baram

**Affiliations:** 1 Department of Pulmonary and Critical Care Medicine, Thomas Jefferson University Hospital, 834 Walnut St Suite 650, Philadelphia, PA 19107 USA; 2 Department of Internal Medicine, Thomas Jefferson University Hospital, 1025 Walnut St Room 805, Philadelphia, PA 19107 USA; 3 School of Population Health, Thomas Jefferson University, 901 Walnut St 10th Floor, Philadelphia, PA 19107 USA; 4 Department of Pathology, Thomas Jefferson University Hospital, 130 South 9th St Room 2109, Philadelphia, PA 19107 USA; 5 Blood Bank and Transfusion Medicine, Thomas Jefferson University Hospital, 111 South 11th St, Philadelphia, PA 19107 USA; 6 Sidney Kimmel Medical College, Thomas Jefferson University, 1025 Walnut St, Philadelphia, PA 19107 USA

**Keywords:** Point-of-care lab testing, Massive transfusion protocol, Hemoglobin, Bleeding, Hemorrhage, Transfusion

## Abstract

**Background:**

Point-of-care (POC) hemoglobin testing has the potential to revolutionize massive transfusion strategies. No prior studies have compared POC and central laboratory testing of hemoglobin in patients undergoing massive transfusions.

**Methods:**

We retrospectively compared the results of our point-of-care hemoglobin test (EPOC®) to our core laboratory complete blood count (CBC) hemoglobin test (Sysmex XE-5000™) in patients undergoing massive transfusion protocols (MTP) for hemorrhage. One hundred seventy paired samples from 90 patients for whom MTP was activated were collected at a single, tertiary care hospital between 10/2011 and 10/2017. Patients had both an EPOC® and CBC hemoglobin performed within 30 min of each other during the MTP. We assessed the accuracy of EPOC® hemoglobin testing using two variables: interchangeability and clinically significant differences from the CBC. The Clinical Laboratory Improvement Amendments (CLIA) proficiency testing criteria defined interchangeability for measurements. Clinically significant differences between the tests were defined by an expert panel. We examined whether these relationships changed as a function of the hemoglobin measured by the EPOC® and specific patient characteristics.

**Results:**

Fifty one percent (86 of 170) of paired samples’ hemoglobin results had an absolute difference of ≤7 and 73% (124 of 170) fell within ±1 g/dL of each other. The mean difference between EPOC® and CBC hemoglobin had a bias of − 0.268 g/dL (*p* = 0.002). When the EPOC® hemoglobin was < 7 g/dL, 30% of the hemoglobin values were within ±7, and 57% were within ±1 g/dL. When the measured EPOC® hemoglobin was ≥7 g/dL, 55% of the EPOC® and CBC hemoglobin values were within ±7, and 76% were within ±1 g/dL. EPOC® and CBC hemoglobin values that were within ±1 g/dL varied by patient population: 77% for cardiac surgery, 58% for general surgery, and 72% for non-surgical patients.

**Conclusions:**

The EPOC® device had minor negative bias, was not interchangeable with the CBC hemoglobin, and was less reliable when the EPOC® value was < 7 g/dL. Clinicians must consider speed versus accuracy, and should check a CBC within 30 min as confirmation when the EPOC® hemoglobin is < 7 g/dL until further prospective trials are performed in this population.

## Background

Time is of the essence when it comes to preventing morbidity and mortality for critically ill patients suffering from massive hemorrhage [1]. POC lab testing has revolutionized patient care by providing actionable information at the bedside in a fraction of the amount of time required for a core laboratory test result to come back. However, the possible tradeoff of POC testing is expediency at the expense of accuracy. Our aim was to examine the utility of POC hemoglobin testing in patients with acute hemorrhage who were undergoing massive transfusion of blood products. Aside from the obvious morbidity and mortality associated with under resuscitation, there are problems with over transfusion including: volume overload, respiratory failure, renal failure, and re-bleeding specifically in the setting of variceal bleeds [2, 3]. Additionally, over transfusion results in substantial waste of limited and expensive resources. Even relatively small discrepancies between POC and core laboratory hemoglobin values can lead clinicians to choose very different treatment plans when resuscitating acutely bleeding patients so it is imperative that these tests be highly correlated if clinical decision are to be made based off of them. The purpose of this study was to compare the hemoglobin results from our institution’s POC lab system and our core laboratory system in patients undergoing massive blood-product transfusion for acute blood loss in order to determine how accurate POC hemoglobin testing has during the resuscitation of acutely hemorrhaging patients. We compared the EPOC® Blood Analysis System (Epocal Inc., Ottawa, Ontario, Canada) to the Sysmex-XE 5000™ core laboratory system (Kobe, Japan). Our goal was to clarify the usefulness of this test, which could potentially facilitate early decision-making based on a reliable test in critically-ill patients with acute blood loss requiring large volume transfusion.

## Methods

### Patient population

This was a retrospective chart review at a single-center, tertiary care hospital in Philadelphia, PA. The study protocol was reviewed and approved by our institution’s institutional review board. All patients undergoing massive transfusion protocol (MTP) between 10/2011 and 10/2017 were identified through the hospital’s electronic medical record system. The hospital definition for MTP was “the administration of at least one blood volume worth of blood, or During an MTP, our blood bank sends 6 units of packed red blood cells (PRBCs), 4 units of fresh frozen plasma (FFP), and 1 unit of platelets (PLTs) per round. These patient’s records were then reviewed to identify patients that had an EPOC® and core laboratory hemoglobin specimen drawn within 1 h of each other on the day of the MTP event. EPOC® and CBC hemoglobin values, as well as the date and time of the blood draws were recorded. Additional data collected included patient’s age, sex, diagnosis, hospital service, location, and disposition at time of discharge.

Our initial sample population included 98 patients that had MTP activation, and an EPOC® and CBC hemoglobin drawn within 60 min of each other. From these 98 patients, a total of 218 lab comparison points were obtained. We determined a time cutoff when the mean differences between EPOC® and CBC samples are most likely due to actual differences between hemoglobin values rather than random fluctuations around a true value. Time intervals were coded into six categories: 0 (0–1 min); 1 (1–5 min); 2 (5–15 min); 3 (15–30 min); 4 (30–45 min); 5 (45–60 min). ANOVA was performed to assess mean differences between EPOC® and CBC samples over time. Results indicated mean differences were statistically significant at specific time points, F (5, 215) = 3.98, *p* < 0.01. A Tukey HSD post hoc analysis further specified that time intervals when mean hemoglobin differences were statistically different from samples drawn during time interval 0 (tests that were drawn at the same time) occurred between 30 and 60 min, *p* < 0.05 respectively. Therefore, further analysis was restricted to only those samples drawn within 30 min of each other. The final sample consisted of a total of 90 individual patients, including 49 cardiac surgery patients, 20 general/trauma surgery patients, and 19 non-surgical patients. Among these patients, 170 lab comparison points were obtained. Table 1 demonstrates the average number of blood products transfused, stratified by each patient population.

**Table 1:** Average units of each blood product transfused for the patients that had EPOC® and CBC hemoglobin tests drawn within 30 min of each other stratified by patient population

0–30 Minutes Group	Cardiac Surgery	General/Trauma Surgery	Non-surgical Procedure	Medical
# of Patients	49	20	6	15
Avg PRBC	17.8	23	16	18.6
Avg FFP	10.8	12.9	8	11.5
Avg Platelets	5.7	5	3.3	4.3
Avg Cryoprecipitate	3.3	2.5	1.5	2.2

### Hemoglobin measurements

The medical center core laboratory analyzed samples using a Sysmex-XE 5000™ automated hematology analyzer in which the hemoglobin was directly determined by absorbance spectrophotometry and the hematocrit was calculated as a function of mean corpuscular volume and red blood cell count [4]. The EPOC® Blood Analysis System directly measured hematocrit using AC conductometry and the hemoglobin was calculated as a function of the measured hematocrit, assuming a normal mean corpuscular hemoglobin concentration (MCV) [5].

### Statistical methods

We defined a “clinically significant difference” between the two tests to be ±1 g/dl based on the a priori opinion of a panel of three critical care physicians that routinely treat patients with hemorrhagic shock (MB, BA, FW). The Clinical Laboratories Improvement Amendments (CLIA) criteria for interchangeability of lab tests states that hemoglobin measurements must be within ±7% of one another to be considered interchangeable tests [6].

The Bland-Altman method was performed to examine agreement between measured EPOC® and CBC hemoglobin values. This method of analysis is useful when comparing new measurements with the “gold standard” and assists with evaluating the degree of agreement or disagreement between measures [7]. Bland-Altman includes identification of bias, for example if the mean difference in EPOC® and CBC measurements lean positive or negative, as well as the establishment of upper and lower limits of agreement (±1.96 SD). Hemoglobin interchangeability between EPOC® and CBC was assessed based on the specified CLIA criteria (±7%). CLIA cutoff values were coded as follows to determine the proportion of hemoglobin values that fell in and outside of CLIA range between zero and 30 min: 0 = difference ≤ 7%; 1 = difference > 7%. EPOC®-CBC hemoglobin differences ±1 g/dl were coded as well to determine the proportion of hemoglobin values within 1 g/dl range or out of range between zero and 30 min: 0 = hemoglobin ≤1 g; 1 = hemoglobin > 1 g. Additional investigation of EPOC® and CBC Interchangeability was assessed based solely on EPOC® measurements of hemoglobin levels. The proportion of hemoglobin values within the ±7% CLIA range, and within the ±1 g/dl clinically significant range between zero and 30 min was calculated. Furthermore, changes in hemoglobin differences were explored according to medical population. The proportion of hemoglobin values ±1 g/dl within 30 min were computed according to cardiac surgery, general surgery, and non-surgery medical populations. Finally, t-tests were conducted to assess statistical significance with mean CLIA measurements, and with mean hemoglobin ±1 g/dl differences when EPOC® measurements are ≤7 and when > 7. ANOVA testing was performed to evaluate mean hemoglobin differences across population types. Statistical Package for the Social Sciences (SPSS) software was utilized to perform all coding and data analysis. Statistical significance for data analysis was determined using alpha level 0.05.

## Results

**Fig. 1:**
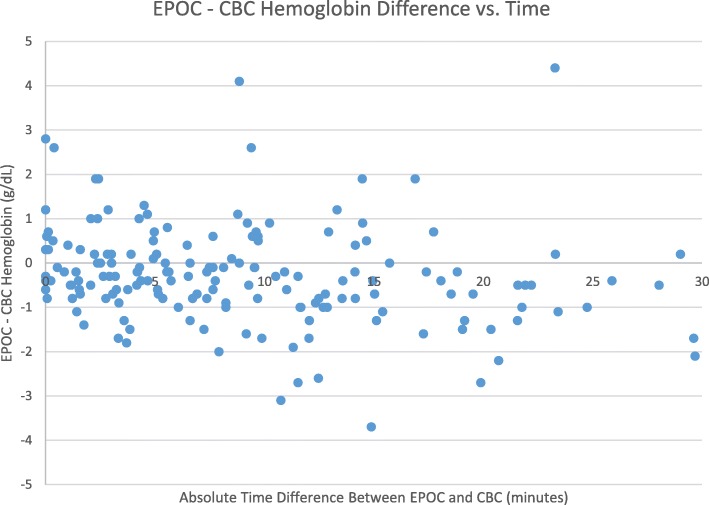
Hemoglobin Difference vs. Time Plot for time 0–30 min. Points that fall within y = 1 and y = − 1 represent clinically insignificant differences between the EPOC® and CBC hemoglobin values

When EPOC® and CBC hemoglobin were drawn within 30 min of each other, 51% (86 of 170) of lab comparison points had an absolute difference of < 7 and 49% (84 of 170) had an absolute difference ≥ 7%. In the same group, 73% (124 of 170) of lab comparison points fell within ±1 g/dL of each other, and 27% (46 of 170) had differences that were > ±1 g/dL (Fig. 1).

**Fig. 2:**
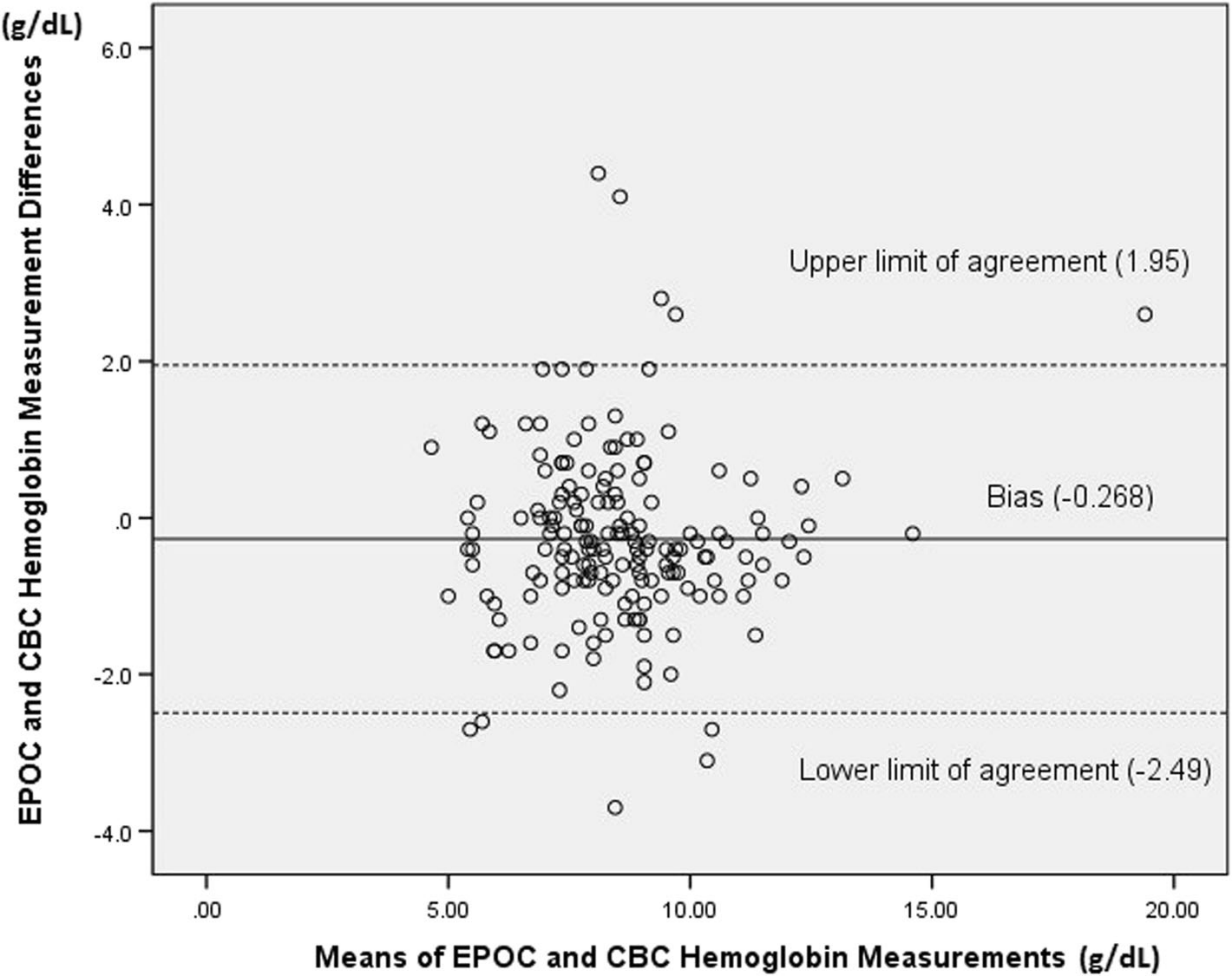
*Bland-Altman Analysis (EPOC*®*-CBC Differences / Measurement Means). Demonstrates a negative bias for EPOC*®

Bland-Altman analysis demonstrated that the mean difference between EPOC® and CBC hemoglobin had a bias of − 0.268 g/dL that was statistically significant, *p* = .002, which indicates that on average, reported EPOC® hemoglobin values were slightly lower than CBC hemoglobin values at each comparison point in our sample (Fig. 2). Although this difference was statistically significant, it was less than our clinically significant difference of ±1 g/dL.

When the measured EPOC® hemoglobin was < 7 g/dL, 30% of the EPOC® and CBC hemoglobin values were within ±7% of one another, and 57% were within ±1 g/dL. When the measured EPOC® hemoglobin was within ≥7 g/dL, 55% of the EPOC® and CBC hemoglobin values were ± 7% of one another, and 76% were within ±1 g/dL. Furthermore, t-tests determined that mean differences between the percent of hemoglobin values within the ±7% range, and the percent of hemoglobin values within the ±1 g/dL range as a function of the measured EPOC® hemoglobin value were statistically significant, 3.01, *p* < 0.01 and − 3.42, *p* < 0.01 respectively.

The EPOC® versus CBC relationships also changed as a function of our patient population breakdown. The difference between EPOC® and CBC hemoglobin was within ±1 g/dL for each population as follows: 77% of the time for cardiac surgery patients, 58% of the time for general surgery patients, and 72% of the time for non-surgical patients. ANOVA analysis determined mean differences in hemoglobin levels among populations (0-30 min) were statistically significant, *F* (2, 167) = 5.051, *p* = 0.007. Post-hoc Tukey’s HSD further revealed mean differences among General Surgery and Cardiac populations. General Surgery populations were statistically significant from both Cardiac and Non-surgical / medical populations, *p* < 0.05 while Cardiac patients were statistically significant from General Surgery patients, *p* < 0.02.

## Discussion

While there are studies that compare POC versus central laboratory parameters in both normal and critically ill patients, there are none to our knowledge that specifically look at hemoglobin values in patients suffering from large volume blood loss requiring massive transfusion [8, 9]. This is a unique, high risk patient population that can experience substantial morbidity and mortality in the setting of imprecise transfusion strategies. POC testing has the potential to revolutionize resuscitation of these patients. It is generally agreed upon among critical care providers, that in the setting of massive blood loss with shock, measured hemoglobin concentrations do not accurately reflect circulating hemoglobin levels, and providers should initially transfuse patients to adequately correct their abnormal vital signs regardless of lab measurements. However, there is a period of time during a resuscitation when a patient’s vital signs have normalized and there is some uncertainty about whether to continue or stop transfusions. Typically there is a delay in getting lab results from central laboratory tests, which could result in patients receiving more or less blood products than they should. At our institution, this delay is on average around 30 min. This key point is where POC hemoglobin testing has potential to improve treatment strategies. Prior studies comparing core versus POC laboratory results in critically ill patients have questioned the reliability of POC hemoglobin measurements. Allardet-Servent et al. compared the POC Siemens RAPIDPoint® 500 blood gas system to their hospital’s central laboratory analyzer in 314 paired samples that were collected prospectively from 51 critically ill patient, and they found that all tested parameters except for hemoglobin satisfied CLIA criteria of interchangeability [8]. The EPOC® device uses conductivity to measure hematocrit [5]. The hemoglobin concentration is calculated from the measured hematocrit with the formula: Hemoglobin (g/L) = Hematocrit (decimal fraction) × 340 [10–12]. Conductometric measurements are affected by changes in temperature, electrolyte concentrations, and protein concentrations within the sample [10, 13]. While conductivity-based hematocrit is considered accurate for most physiologically normal patients, its accuracy is affected by changes in sodium levels, changes in protein concentrations, administration of crystalloid and colloid volume expanders, cell saver transfusions, anticoagulants used in extracorporeal membrane oxygenation (ECMO) and cardiopulmonary bypass for cardiac surgery, and the presence of elevated white blood cell counts, which were common in our patient population [10, 13]. This method also assumes a normal mean corpuscular volume (MCV) concentration, which can lead to inaccuracy since it is possible for normal healthy subjects to have MCV values that fall outside the normal range [14, 15]. Maslow et al. evaluated three different POC devices in 24 patients undergoing cardiac surgery. They demonstrated that POC hemoglobin and hematocrit measurement bias, while although low and technically acceptable, it varied as a function of the hemoglobin level and as the clinical context changed, for example intraoperatively versus postoperatively [16]. Chen et al. conducted a head to head comparison of the EPOC® device to a central lab analyzer as well as number of other commercially available POC devices in patients undergoing cardiopulmonary bypass [10]. The EPOC® conductometrically measured hemoglobin was compared to a co-oximetrically measured hemoglobin. They reported a bias of only 2.78%, which was surprisingly lower than all other POC devices they evaluated, even with the hematocrit result range varying widely from 24 to 45% [10]. These authors did not have an explanation for why the bias was so much lower for the EPOC® device’s hemoglobin in their study, especially since it was not consistent with the results of the other POC devices they had tested which utilized similar technology or prior literature that demonstrated that other POC devices using conductometric hemoglobin measurements were not that accurate in similar patient populations [10, 17–19].

We performed a retrospective study comparing point of care measurements of hemoglobin to central lab testing of hemoglobin during massive transfusion protocols. Our purpose was to determine the clinical utility, reliability and interchangeability of POC measurements during MTP. We were able to demonstrate that in a population of acutely bleeding patients undergoing massive blood-product transfusion, the EPOC® and central laboratory hemoglobin tests only fulfill CLIA criteria for interchangeability 51% of the time. This CLIA criteria is quite rigid and therefore did not represent clinical utility in our assessment. Our clinical experts concluded that clinical utility was indeed achieved with the EPOC device to within ±1 g/dL of hemoglobin. The EPOC® and core laboratory tests were within our clinically significant range 73% of the time suggesting that the EPOC® hemoglobin test has clinical utility. For patients with an EPOC® hemoglobin result of < 7 g/dL, the EPOC® hemoglobin results were more likely to be significantly discrepant from the core laboratory CBC hemoglobin results, whereas patients with an EPOC® hemoglobin result ≥7 g/dL were the most closely correlated with their corresponding CBC results. This discrepancy might be explained by the rapidity of infused products when hemoglobin levels are < 7 g/dL as compared to when the hemoglobin levels are ≥7 g/dL.

There are several limitations to our study. This was a retrospective review of a heterogeneous population of bleeding patient requiring massive transfusion. Because this was a retrospective study, not all of our samples were collected at the exact same time, and although this makes it more of a real-world type scenario, it allows for bias from actual differences in hemoglobin values at different times to affect our results. We arbitrarily chose 60 min to be the cutoff for our inclusion criteria to increase our sample size. As a result there was potential that differences between EPOC® and CBC hemoglobin values were actually due to differences between the two hemoglobin values at different points in time, and not just from intrinsic variability between the lab tests given that these patients were actively bleeding, receiving large volumes of colloid and crystalloid, and various other medications and interventions that could all potentially affect hemoglobin values. We attempted to account for this by limiting our analyzed population to those who had tests drawn within 30 min of one another based on statistical analysis that demonstrated statistically insignificant differences between these time points. Furthermore, the 4PRBC: 4FFP: 1PLT ratio of blood products in our MTP is the standard release of products from the blood bank; however, it is up to the provider which products are actually given and when. This means there was no standardized timing of hemoglobin checks, and this was purely at the discretion of the provider. This made standardization of timing differences between central lab testing and POC testing unreliable. Finally, this study was performed at a single-center with a small sample size.

The results of this study are warranted for our institution’s procedures and devices, and should be interpreted with caution at others institutions. A prospective trial comparing POC and core laboratory hemoglobin values sampled at the same time in acutely hemorrhaging patients undergoing massive blood-product transfusion is needed in order to accurately assess the utility of POC hemoglobin testing in this patient population.

## Conclusions

Similar to previously published data, our study demonstrates that there are limitations to the accuracy of point-of-care hemoglobin testing. Specifically, we demonstrated that in critically-ill bleeding patients requiring massive transfusion of blood products, the EPOC® hemoglobin test was only within the interchangeability range of ±7% with our central laboratory’s measurement 51% of the time. However, the hemoglobin values fell within our clinically acceptable range of ±1 g/dL 73% of the time which suggests utility for this point-of-care test in hyperacute situations during which clinicians may favor the rapidly available POC hemoglobin results with a margin of error within ±1 g/dL rather than wait an excess of 30 min for the central lab test result. Anecdotally, critical care providers at our institution report that point-of-care hemoglobin testing has resulted in both miraculous saves and inappropriate over- and under-resuscitation of bleeding patients. Our current data, though limited, support the idea that there is utility for the EPOC® hemoglobin test, but that it has limitations in this specific patient population. Therefore, providers must continue to weigh the risks and benefits of utilizing point-of-care hemoglobin testing on a case-by-case basis until larger prospective studies are performed in this unique patient population. Future studies should be prospective comparing both testing methods of hemoglobin sampled at the same time.

## Data Availability

The datasets during and/or analyzed during the current study are available from the corresponding author on reasonable request.

## Abbreviations

CBC: Complete blood count
CLIA: Clinical Laboratory Improvement Amendments
ECMO: Extracorporeal membrane oxygenation
FFP: Fresh frozen plasma
MCV: Mean corpuscular volume
MTP: Massive transfusion protocol
PLT: Platelets
POC: Point-of-care.
PRBC: Packed red blood cells
